# Effects of osteoprotegerin from transfection of pcDNA3.1(+)/chOPG on bioactivity of chicken osteoclasts

**DOI:** 10.1186/1751-0147-53-21

**Published:** 2011-03-24

**Authors:** Lele Hou, Jiafa Hou, Jing Yao, Zhenlei Zhou

**Affiliations:** 1 College of Veterinary Medicine, Nanjing Agricultural University, Nanjing, Jiangsu, 210095, China

## Abstract

**Background:**

Osteoprotegerin (OPG) has been reported to prevent bone resorption by inhibiting the formation, function, and survival of osteoclasts in a variety of animal models. However, the effects of OPG on bone metabolism in avian species have not been described. The objective of this study was to investigate the effects of chicken OPG (chOPG) expressed in chicken embryo fibroblasts (CEFs) on chicken osteoclast function *in vitro*.

**Methods:**

The chOPG sequence containing the open reading frame (ORF) was amplified from chicken embryo frontal bone and inserted into the pcDNA3.1 (+) vector. PcDNA3.1 (+)/chOPG was transiently transfected into CEFs by lipofectamine 2000. Transcription of OPG mRNA and expression of chOPG recombinant protein were detected by reverse transcription polymerase chain reaction (RT-PCR) and indirect immunofluorescence. The level of chOPG recombinant protein was detected by enzyme-linked immunosorbent assay. The suspension of osteoclasts was separated from chicken embryos and divided into three groups (control group, pcDNA3.1 (+) group and pcDNA3.1 (+)/chOPG group). The percentage of osteoclast apoptosis was detected by flow cytometry. The tartrate-resistant acid phosphatase (TRAP) secreted by osteoclasts was measured by the diazol method. The resorbing activity of osteoclasts was evaluated by the area of lacunae on bone flaps and the concentration of calcium in the supernatant liquid of osteoclasts.

**Results:**

48 h after transfection, the exogenous OPG gene transcription was detected by RT-PCR. After 72 h, the CEFs transfected from pcDNA3.1 (+)/chOPG displayed green fluorescence and the concentration of chOPG protein was 15.78 ± 0.22 ng/mL. After chicken osteoclasts were cultured for 5 d in a medium containing supernatant from transfected CEFs, the percentage of osteoclast apoptosis was increased significantly, the concentration of TRAP, the area of lacunae on bone flaps and calcium concentration were decreased significantly in the pcDNA3.1(+)/OPG group compared to the control group and the pcDNA3.1 (+) group.

**Conclusion:**

Constructed pcDNA3.1 (+)/chOPG transfected into CEFs expressed bioactive OPG protein that was able to inhibit osteoclast function.

## Background

Osteoporosis in laying hens is a condition that involves a progressive loss of bone resulting in bone fragility and increased risk of fracture. Surveys of laying flocks in Europe have indicated that about 30% of the birds experience one or more bone fractures due to osteoporosis during their lifetime. The high fracture rates show that osteoporosis not only leads to production losses, but also to severe welfare problems in hens [1].

In laying hens, the main types of bone providing structural integrity are cortical and trabecular bone. In addition to these, medullary bone, an extremely labile source for calcium that develops in specific bones of female birds at the onset of sexual maturity, provides a labile source of calcium for shell formation. Bones undergo a constant process of remodelling, which at the cellular level involves a coordinated regulation of osteoblasts and osteoclasts. As hens mature sexually, bone formation of osteoblasts switches from structural bone to medullary bone [2]. In the absence of structural bone formation, continued osteoclastic resorption of structural bone will result in a depletion of structural bone, ultimately leading to osteoporosis.

The differentiation and function of osteoclasts are regulated by soluble cytokines from osteoblasts, such as osteoprotegerin (OPG) and the receptor activator of nuclear factor ligand (RANKL; also called OPG ligand) [3]. OPG is a soluble decoy receptor that inhibits osteoclast formation, function, and survival by preventing the binding of RANKL to the receptor activator of nuclear factor κB (RANK), a membrane-bound protein that is found on chondrocytes, dendritic cells, osteoclast precursors, and mature osteoclasts [4]. Many cytokines and effectors are known to influence the osteoclastic bone resorption via the OPG/RANK/RANKL trio of proteins [5,6]. Changes of expression levels of OPG/RANK/RANKL would be expected to cause bone disorders such as postmenopausal osteoporosis, glucocorticoid-induced osteoporosis, and sporadic Paget's disease in man [7].

Although the importance of OPG in the osteoclastogenesis has been established in mammalian models, it is not yet clear how OPG regulates the function of osteoclasts in avian species. To elucidate the function of OPG in laying hens *in vivo*, we amplified the open reading frame (ORF) of the chicken OPG (chOPG) sequence, constructed the pcDNA3.1 (+)/chOPG plasmid and transiently transfected it into chicken embryo fibroblasts (CEFs). We tested whether pcDNA3.1 (+)/chOPG expressed OPG protein at a level able to inhibit the biological activity of osteoclasts *in vitro*.

## Methods

### Cloning of the ORF of chOPG

Total RNA was extracted from chicken embryonic frontal bone (Animal Husbandry Industry Co., Nanjing, China) with TRIzol^® ^Reagent (Invitrogen, Inc. Carlsbad, CA, USA) according to the manufacturer's instruction. RNA purity was determined by 260 nm and 280 nm absorbance ratios and integrity was checked by 1% agarose/formaldehyde gel electrophoresis. A Biometra DNA Thermal Cycler was used for reverse transcription polymerase chain reaction (RT-PCR). RT-PCR was performed in the presence of DTT, oligo(dT)18, dNTP, RNase inhibitor, first-strand buffer and Moloney murine leukaemia virus reverse transcriptase (TakaRa Bio Inc. Japan). The final mixture was reacted at 42°C for 60 min and at 70°C for 15 min to denature the enzyme. On the basis of the published nucleotide sequence of chOPG (DQ098013), one pair of PCR primers (Invitrogen) were designed. Primers P1 and P2 were used to amplify the ORF of chOPG sequence. *Nhe*| and *Xho*| (TakaRa Bio Inc.) restriction sites were inserted into primers P1 and P2, respectively:

P1: 5'-CAT*GCTAGC*ATGAACAAGTTCCTGTGC-3' (sense strand, positions 10-27 of cDNA sequence);

P2: 5'-CCGG*CTCGAG*TTAGACACATCTTACTTT-3' (antisense strand, positions 1,201-1,218 of cDNA sequence).

PCRmix (TakaRa Bio Inc.), primers P1 and P2, and cDNA were mixed and amplified for 30 cycles under the following conditions: denaturation for 30 s at 94°C, annealing for 45 s at 47°C, and extension for 50 s at 72°C. The products were subsequently sequenced (Invitrogen) after 1% agarose electrophoresis, recovery and purification.

### pcDNA3.1 (+)/chOPG construction

The eucaryote expression vector pcDNA3.1 (+) (Invitrogen) and OPG products were digested with *Nhe*| and *Xho*|. After purification, two fragments were ligated with T4DNA ligase (TakaRa Bio Inc.) at 16°C (overnight). The ligation product was subsequently transformed into DH5α competent cells (Nanjing Agricultural University, Nanjing, China)). The transformed cells were plated on Luria-Bertani agar (Invitrogen-Gibco, Grand Island, NY, USA) containing ampicillin (Invitrogen-Gibco). The positive clones were identified by PCR (PCRmix, pcDNA3.1 (+) consensus primer P3 (5'-CTGGCTAACTAGAGAACCCAC-3'), P4 (5'-TAGAAGGCACAGTCGAGG-3')). DNA of positive clones were mixed and amplified for 30 cycles under the following conditions: denaturation for 30 s at 94°C, annealing for 45 s at 49°C, and extension for 50 s at 72°C and double restriction digestion, followed by agarose gel analysis. Then pcDNA3.1 (+)/chOPG and pcDNA3.1 (+) were prepared with non-endotoxin plamid extraction kit (Sigma Chemicals. St. Louis, MO, USA).

### Cell culture and DNA transfection

CEFs were prepared from two 10-days old chicken embryos (Animal Husbandry Industry Co) and were grown according to standard procedures, cultured in Dulbecco's modified Eagle's medium (DMEM) (Invitrogen-Gibco) supplemented with 5% fetal bovine serum (Invitrogen-Gibco). The number of cells was adjusted to 2 × 10^5 ^cells/ml and incubated in 24-well tissue culture plates (Bo Quan Sci&Tech. Co. Ltd. Nanjing, China) containing cover glass at 37°C in a humid atmosphere of 5% CO_2 _for 24 h. Prior to each test, CEFs were washed three times with phosphate buffered solution (PBS), transfected with 1 μg/well pcDNA3.1 (+)/OPG plasmid and pcDNA3.1 (+) vector using 3 μl/well lipofectamine 2000 (Invitrogen), respectively, followed by incubation at 37°C in 5% CO_2 _for 48 h and 72 h. The culture medium was renewed every 2nd day.

### RT-PCR analysis of chOPG mRNA

The cells (both floating and adherent cells) were harvested 48 h post transfection. The total RNA was extracted with TRIzol^® ^Reagent according to the manufacturer's instruction. RNA samples were then treated with DNase I (1 U/μg) (TakaRa Bio Inc.) before the RT step to avoid the interference with contaminating genomic DNA. P5 (5'-ATGAACAAGTTCCTGTGC-3') and P6 (5'-TTAGACACATCTTACTTT-3') were subjected to PCR using upstream and downstream primers.

### Immunocytochemical analysis of chOPG product in CEFs

CEFs (2 × 10^5^) cultured for 72 h on glass coverslips (6 mm × 6 mm) were replated into 24-well plates. Glass coverslips were washed with 0.01 M PBS and fixed in 4% formaldehyde for 45 min. Detergent extraction with 3% Triton X-100 was performed for 10 min. Coverslips were saturated with PBS containing 5% bovine serum albumin (Wuhan Boster Biotechnology Company, China) for 1 h at room temperature with gentle rocking, processed with rabbit anti-chOPG polyclonal antibody (Nanjing Agricultural University) for 1 h at 37°C and followed by FITC-goat-anti-rabbit IgG (Wuhan Boster Biotechnology Company) for 1 h at 37°C and then stained by DAPI staining solution (Wuhan Boster Biotechnology Company). Coverslips were washed by PBS for 30 min prior to each treatment. Finally, coverslips were mounted on slides and fluorescence signals were analyzed by a Fluoview microscopy (Olympus, Japan).

### ELISA analysis of chOPG product in supernatant

The concentration of the chOPG product in the supernatant was determined using an enzyme-linked immunosorbent assay (ELISA) kit (R&D Systems, Minneapolis, MN, USA) according to the manufacturer's instructions. The concentration was determined for three wells of each sample by measuring the optical density (OD) at 450 nm wavelength by an ELISA reader (Immuno Mini NJ-2300, InterMed, Japan).

### Effect of chOPG on osteoclast bioactivity

Tibias and humeri were isolated from 15 18-days old chicken embryos. Osteoclast cultures were prepared as previously described [8]. Briefly, a cell suspension was seeded at a concentration of 2 × 10^5 ^cells per well in 24-well dishes containing either glass coverslips or bovine bone slices (4 mm × 4 mm × 50 μm) (Nanjing Agricultural University). Non-adherent cells were washed off after 2 h. The adherent cells were grown for another 2 d and then cultured in DMEM containing OPG supernatant. The medium was changed every 48 h. Glass coverslips, bovine bone slices and supernatant were harvested after 5 d. The percentage of osteoclast apoptosis was detected by flow cytometry. TRAP secreted to the supernatant by osteoclasts was measured at a OD of 530 nm by the diazol method using TRAP test kit (Bo Quan Sci&Tech. Co. Ltd. Nanjing, China). The resorption lacunae on the bone slice was visualized by toluidine blue staining after removal of osteoclasts using 50 mM NH_4_OH and brief sonication [9]. The concentration of calcium in the supernatant was determined by atomic absorption spectrometry (wavelength 422.7 nm, electric current 3.0 mA, spectrum width 0.4 nm) after 5 times dilution.

### Statistical analysis

All values were expressed as means ± the standard deviation (SD). Differences between mean values of normally distributed data were assessed by the one-way ANOVA test and Student's *t*-test. Statistical difference was accepted at *P *< 0.05.

## Results

### Cloning of the ORF of chOPG and construction pcDNA3.1 (+)/chOPG

**Figure 1:**
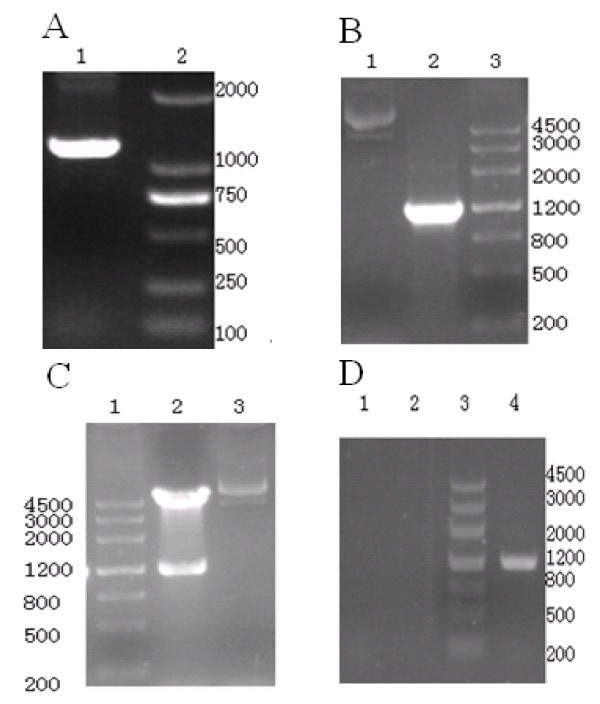
**Gel electrophoresis of chOPG. 1A**: Gel electrophoresis of reverse transcription polymerase chain reaction (RT-PCR) product. Total RNA extracted from chicken embryo frontal bone was analyzed using RT-PCR with specific primers. About 1.2 kbp gene of chicken osteoprotegerin (chOPG) was amplified (lane 1); DL2000 marker (lane 2); **1B**: Gel electrophoresis of pcDNA3.1 (+)/chOPG PCR product. chOPG fragment was inserted into the eucaryon expression vector pcDNA3.1 (+) between *Nhe*| and *Xho*|. Negative plasmid (lane 1) and positive plasmid (lane 2) were chosen using PCR; marker (lane 3); **1C**: Gel electrophoresis of pcDNA3.1 (+)/chOPG double restriction enzyme assay. Positive plasmid (lane 3) was identified by *Nhe*|and *Xho*|double restriction digestion and showed pcDNA3.1 (+) and OPG (lane 2); marker (lane 1); **1D**: Gel electrophoresis of RT-PCR analysis showing the expression of chOPG gene at 48 h. Amplification of chOPG using cDNA from lane 1 (control group) and lane 2 (pcDNA3.1 (+) transfected CEFs group) showing negative result. Amplification of chOPG using cDNA from lane 4 (pcDNA3.1 (+)/chOPG transfected chicken embryo fibroblasts group) showing about 1200 bp gene of chOPG. Lane 3: marker

The size of the specific gene fragment amplified was, as expected, about 1.2 kbp (Figure 1A). The positive clones were identified by PCR amplification and the double restriction digestion with *Nhe*| and *Xho*| (Figure 1B and Figure 1C). Analysis of the PCR products by agarose gel electrophoresis showed that both constructs contained a DNA insert of the correct size and in the correct orientation. The result of sequencing showed that it had 100% homology with that reported in GenBank (DQ098013) indicating that the *OPG *gene has an extensive hereditary conservation and that no mutations were present in this region of the vector.

### Expression of chOPG in CEFs transfected with pcDNA3.1 (+)/chOPG

RT-PCR analysis indicated that CEFs in the group with pcDNA3.1(+)/chOPG transfection expressed OPG mRNA, but there was no expression of OPG mRNA in the control group and pcDNA3.1(+) group (Figure 1D).

**Figure 2:**
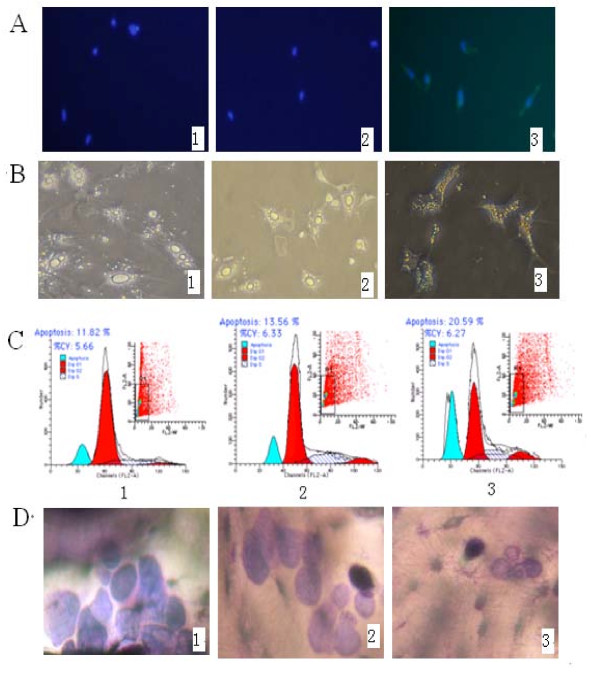
**The expression of chOPG protein and effect on osteoclast morphology, apoptosis and resorption. 2A**: Immunofluorescence assay for a possible chicken osteoprotegerin (chOPG) protein. Chicken embryo fibroblasts (CEFs) were grown on coverslips, fixed, and examined by indirect immunofluorescence. Cells were incubated with rabbit anti-chOPG serum. The secondary antibody was fluorescein-conjugated goat anti-rabbit immunoglobulin G (green). The nuclei of the corresponding cells were visualized by DAPI staining (blue). Fluorescence signals were analyzed by Fluoview microscopy (×200). Negative results are shown on card l (control group) and card 2 (pcDNA3.1 (+) transfected CEFs group), positive green fluorescence for CEFs are shown on card 3 (pcDNA3.1 (+)/chOPG transfected CEFs group). **2B**: The morphology of osteoclasts was observed by inverted phase contrast microscope (×200). The adherent osteoclasts were cultured in Dulbecco's modified Eagle's medium (DMEM) containing supernatant of control group (l), pcDNA3.1 (+) transfected CEF group (2) and pcDNA3.1 (+)/chOPG transfected CEF group (3) for 5 d. **2C**: Effect of the supernatant of three groups on the apoptosis of osteoclasts by flow cytometry. **2D**: Toluidine blue staining of bone slices showing resorption lacunae (×200). The adherent osteoclasts were cultured in DMEM containing supernatant for 5 d in three groups.

Immunofluorescence studies showed that chOPG protein was distributed in the cytoplasma and CEFs showing green fluorescence were observed in the pcDNA3.1 (+)/chOPG group, but were not present in the other groups (Figure 2A).

In the culture supernatant of the pcDNA3.1 (+)/chOPG group transfected from CEFs, the concentration of chOPG was 15.78 ± 0.22 ng/ml, whereas chOPG was not be demonstrated in media from the control group or the pcDNA3.1 (+) group.

### Effect of product from transfected CEFs on chicken osteoclast bioactivity in vitro

**Figure 3:**
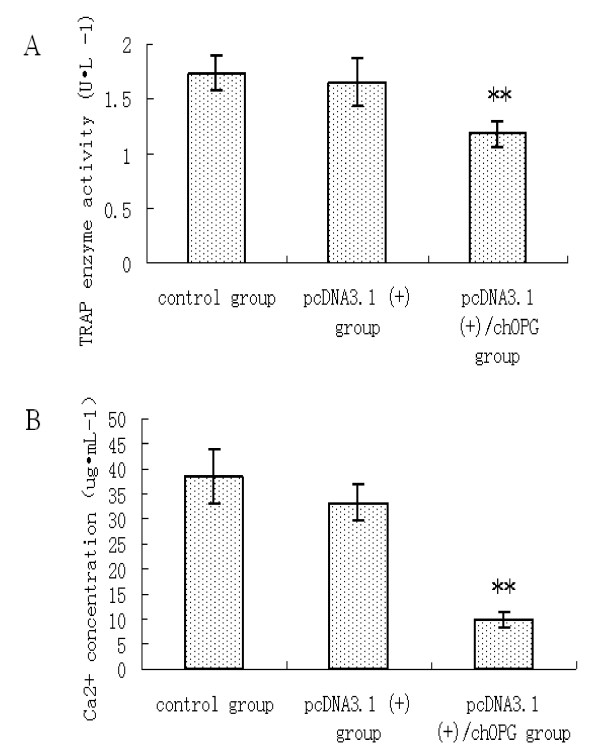
**The change of TRAP enzyme activity and concentration of Ca2+ in three groups. 3A**: Effect of culture supernatant from chicken embryo fibroblasts transfected on osteoclastic TRAP enzyme activity (Mean ± SD; n = 8). ** indicates *P *< 0.01 compared with the control group. **3B**: The concentration of Ca^2+ ^in the supernatant containing bovine bone slices (Mean ± SD; n = 8). ** indicates *P *< 0.01 compared with the control group.

The morphology of osteoclasts after culturing for 5 d is shown in Figure 2B. Osteoclasts grew well in the control and pcDNA3.1 (+) transfected CEF groups, whereas major nuclei disappeared, many vacuoles and lipid droplet appeared in the cytoplasm and many non-adherent and dead osteoclasts were observed in the culture solution of the pcDNA3.1 (+)/chOPG transfected CEF group. The percentage of osteoclast apoptosis in the control, pcDNA3.1 (+) and pcDNA3.1 (+)/chOPG groups was 10.32%±1.50%, 12.61%±0.95%, 20.59%±2.83%, respectively (Figure 2C). TRAP enzyme activity in the pcDNA3.1 (+)/chOPG group was significantly decreased compared to the control group (*P *< 0.01) (Figure 3A). An individual resorption event was seen as a dark border of toluidine blue stain surrounding an excavation. The data were recorded for each resorption event separately (Figure 2D). The quantity and area of lacunae reflected bone resorption by osteoclasts (Table 1). DMEM culture solution did not contain Ca^2+ ^until after culturing thus suggesting osteoclast activity (Figure 3B).

**Table 1:** Effect of culture supernatant of chicken embryo fibroblasts on the quantity and area of osteoclast resorption lacunae in three groups.

	***control group***	***pcDNA3.1 (+) group***	***pcDNA3.1 (+)/chOPG group***
Number of lacunae	10.7 ± 1.2	9.0 ± 1.0	5.4 ± 0.5
Areas of lacunae (μm2)	5755.2003 ± 234.7778	4987.7468 ± 124.5471	739.4407 ± 150.1978**

Note: compared with the control group, ** *P *< 0.01

## Discussion

Bone is an exceedingly complex tissue with multisystemic regulation. Skeletal metabolism depends on the dynamic balance of bone formation by osteoblasts and bone resorption by osteoclasts. The discovery of the OPG/RANKL/RANK system in the mid 1990s has led to major advances in our understanding of how bone modeling and remodeling are regulated [10]. Current research has focused on OPG in humans and mice, while reports on avian OPG are lacking. In our laboratory, chOPG mRNA was extracted from chicken embryo frontal bone. The OPG coding region was successfully amplified and sequence analysis indicated that OPG is highly conserved evolutionary. The sequence reported here had a 68.76%, 68.60% and 68.29% homonology to human, rat and mouse OPG, respectively. The sequence similarity suggests a similar function across species.

Bone is particularly intriguing in laying hens because of the huge demands for calcium for eggshell formation and the occurrence of medullary bone. On the surface of the medullary bone, osteoclasts undergo cyclical functional modifications during the egg-laying cycle [11].

In this study, chOPG induced osteoclast apoptosis after *in vitro *incubation for 5 d. This result was similar to that reported by Lacey *et al*. [12], who demonstrated that OPG inhibited bone resorption and induced osteoclast apoptosis though inhibition of F-actin ring formation of mature osteoclasts or altered interaction between stroma cell and osteoclasts.

The results suggest that the secretion of TRAP by osteoclasts was significantly decreased; further demonstrating that recombinant chOPG could inhibit the activity of osteoclasts *in vitro*. Chamber *et al*. [13] and Boyde *et al*. [14] provided evidence for a direct association between the quantity, area and depth of absorption and the capability of osteoclasts to resorption bone. The present study showed that chOPG inhibited osteoclast bone resorption and consequently the concentration of Ca^2+ ^in the supernatant was significantly reduced. However, the mechanisms by which OPG exerts its biological activity as well as the nature of its molecular interactions with osteoclasts are not well defined. Hakeda *et al*. [15] reported the first evidence of a direct biological activity of OPG on isolated osteoclasts via a 140 kDa OPG-binding protein. The exact nature of osteoclastic OPG receptors was not further characterized. Direct biological activities of OPG on osteoclasts were recently showed by Wittrant *et al*. [16] demonstrating OPG enhanced proMMP-9 activity along with several other parameters (TRAP, TIMP, cathepsin K) in purified osteoclasts. Theoleyre *et al*. [17] showed that OPG stimulates proMMP-9 activity of osteoclasts by the ras/MAPK pathway involving p38 and ERK1/2 phosphorylations. Moreover, OPG-induced MAPK pathway depends on RANKL. In general, OPG is not only a soluble decoy receptor for RANKL as described in the literature but may be also considered as a direct effector of osteoclast functions.

## Conclusions

ChOPG is capable of inhibiting bone resorption as well as promoting osteoclast apoptosis. The study also indicates that pcDNA3.1 (+)/chOPG may be a target for regulating bone metabolism in chicken bone metabolic diseases such as osteoporosis.

## Abbreviations

CEFs: chicken embryo fibroblasts; chOPG: chicken OPG; DMEM: Dulbecco's modified Eagle's medium; OD: optical density; OPG: osteoprotegerin; ORF: open reading frame; PBS: phosphate buffered solution; RANKL: receptor activator of nuclear factor κB ligand; RANK: receptor activator of nuclear factor κB; RT-PCR: reverse transcription polymerase chain reaction; TRAP: tartrate-resistant acid phosphatase.

## Competing interests

The authors declare that they have no competing interests.

## Authors' contributions

LH and JH conceived of the study, and participated in its design and coordination and helped to draft the manuscript. ZZ participated in the data collection. JY cultured the chicken embryo osteoclasts. LH performed the other experiments. All authors have been involved in drafting the manuscript and have read and approved the final manuscript.
